# Current Evidence on the Relation Between Microbiota and Oral Cancer—The Role of *Fusobacterium nucleatum*—A Narrative Review

**DOI:** 10.3390/cancers17020171

**Published:** 2025-01-07

**Authors:** Federica Chiscuzzu, Claudia Crescio, Simona Varrucciu, Davide Rizzo, Michela Sali, Giovanni Delogu, Francesco Bussu

**Affiliations:** 1 Mater Olbia Hospital, 07026 Olbia, Italy; federica.chiscuzzu@materolbia.com (F.C.); giovanni.delogu@unicatt.it (G.D.); 2 Otolaryngology Division, Azienda Ospedaliera Universitaria di Sassari, 07100 Sassari, Italy; drizzo@uniss.it (D.R.); fbussu@uniss.it (F.B.); 3 Department of Medicine Surgery and Pharmacy, Sassari University, 07100 Sassari, Italy; simonavarrucciu@gmail.com; 4 Dipartimento di Scienze Biotecnologiche di Base, Cliniche Intensivologiche e Perioperatorie-Sezione di Microbiologia, Università Cattolica del Sacro Cuore, 00168 Rome, Italy; michela.sali@unicatt.it; 5 Department of Laboratory and Infectivology Sciences, Fondazione Policlinico Universitario A. Gemelli IRCCS, 00168 Rome, Italy

**Keywords:** oral microbiota, oral cancer, OSCC, CRC, *Fusobacterium nucleatum*, carcinogenesis, adhesins, prognostic factor

## Abstract

Oral squamous cell carcinoma (OSCC) is one the most prevalent head and neck cancers and represents a major cause of morbidity and mortality worldwide. The main established risk factors for OSCC include tobacco and alcohol consumption and betel quid chewing, which may contribute alone or in combination with other environmental factors to carcinogenesis. The oral microbiota is emerging as a key player in the establishment of the molecular and cellular mechanisms that may trigger or promote carcinogenesis, including in the oral cavity. Among the bacterial species found in the oral microbiota, *Fusobacterium nucleatum*, an anaerobic bacterium commonly found in oral biofilms and a periodontal pathogen, has gained attention due to solid evidence implicating *F. nucleatum* in colorectal cancer (CRC). *F. nucleatum* has been shown to induce chronic inflammation, promote cell proliferation and trigger cellular invasion while deploying immune evasion mechanisms. These experimental findings were first obtained in in vitro and in vivo models of CRC and are being confirmed in studies on OSCC. In this review, we summarize the most recent findings on the role of *F. nucleatum* in OSCC, discuss the clinical implications in terms of prognosis and provide an overview of the key mechanisms involved. Moreover, we identify research questions and aspects that require investigations to clarify the role of *F. nucleatum* in OSCC. We anticipate that studies in this emerging field may have a significant clinical impact on the diagnosis, prognosis and management of OSCC.

## Simple Summary

The microbiota and *Fusobacterium nucleatum* (*Fn*) in particular are potential actors in oral oncology. The evidence about the role of *Fn* and the potential underlying mechanisms are reviewed on the basis of the more robust evidence concerning colorectal cancer. The translational potential of the different scenarios linking *Fn* to oral squamous cell carcinoma is discussed.

## 1. Introduction—Epidemiology and Clinical Aspects

**Figure 1:**
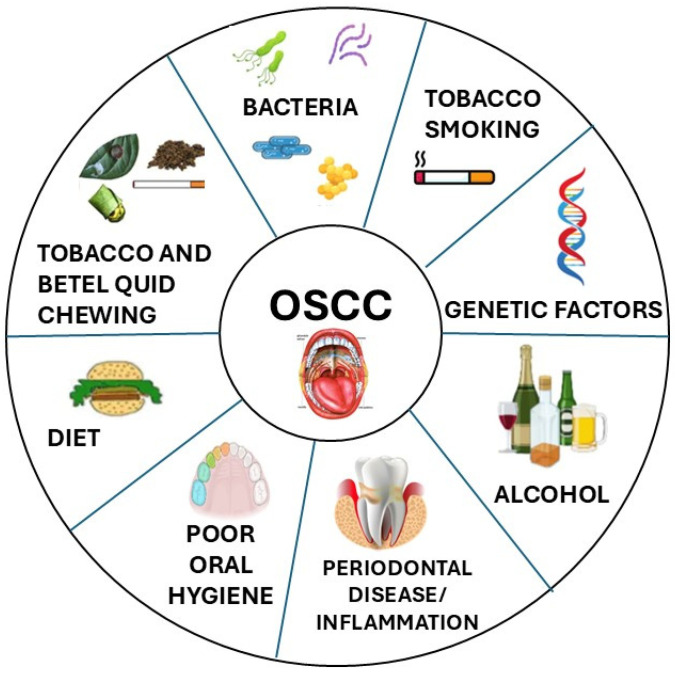
The most likely risk factors contributing to OSCC carcinogenesis.

Head and neck cancers are a group of tumors that generally includes epithelial malignancies arising on the mucosal surfaces of the upper aerodigestive tract (nose and paranasal sinuses, pharynx, larynx, oral cavity) and within the associated major salivary glands (parotid, submandibular and sublingual) [1,2]. Head and neck cancers are themselves very heterogeneous in terms of clinical history, prognosis and responsivity to the main different treatment modalities (surgery, irradiation, chemotherapy and, more recently, immunotherapy) [3,4]. The three main variables defining relatively homogeneous groups of head and neck cancers are the histotype, the primary site and subsite of origin and, for nasopharynx and oropharynx carcinomas, possible virus-induced carcinogenesis (EBV for nasopharynx, HPV for oropharynx) [5]. Among such homogeneous groups, the most common are the squamous cell carcinomas (SCCs) of the larynx (with different features in the different subsites, such as the glottis and the supraglottis), the SCCs of the oropharynx (OPSCCs) and the SCCs of the oral cavity (OSCCs). These three cancers have specific features and highly variable incidence in different areas of the world based on genetic predisposition, but even more on different exposures to carcinogenetic agents (tobacco smoking, tobacco and betel chewing, alcohol abuse, HPV infection) (Figure 1) [2,3].

In the last decades, OPSCC has attracted significant attention because in the US, in relation to what has been defined as an HPV epidemic, it has become by far the most common head and neck cancer with a still-increasing incidence, and it is currently the most common virus-related malignancy [6]. OPSCC is the only head and neck cancer where the carcinogenic and prognostic role of HPV has been demonstrated, and the definition of HPV-driven carcinogenesis has become a mandatory part of the work up, as HPV-induced OPSCCs are now considered a different disease with a different prognosis, response to treatment and UICC/AJCC staging [7,8]. HPV detection in OPSCC is currently, together with the detection of PDL-1 in recurrent/metastatic cases, the only molecular parameter included in all the main head and neck oncology guidelines [1].

Outside the US, for most of the world’s population, where anti-smoking campaigns have not been so successful and, on the other hand, the rate of HPV-induced OPSCC is much lower, OSCC and LSCC remain the most common head and neck cancers [9,10]. For these diseases, HPV has not gained a definite carcinogenic role, and the main risk factors remain smoking and alcohol [11,12]. As for OSCC carcinogenesis, trauma from poorly treated teeth or improperly adapted dentures and, mainly in the Indian subcontinent, tobacco and/or betel chewing, also play a role. As a matter of fact, no molecular parameter currently helps in defining the prognosis and treatment choice in daily clinical practice, neither for LSCC nor for OSCC. TNM staging and “classical” histopathological adverse features (such as perineural invasion, depth of invasion and extranodal spread) remain the only tools to predict the prognosis and to modulate treatment in LSCC and OSCC, but they appear largely insufficient as tumors apparently similar under a histopathological point of view may notoriously display drastically different clinical behaviors [1,2,4]. OSCC has an overall slightly worse prognosis than LSCC and OPSCC, and it is typically radio-chemoresistant so that the preferred treatment modality is surgery in most guidelines. The means to predict the clinical behavior and in particular the nodal spread, which is probably the single most relevant parameter under an oncological point of view, may bring a significant benefit for the management of OSCC. HPV infection raised great enthusiasm in relation to this aim in the last decades, but the findings about its role have always been conflicting [13], and recent studies exclude any relevant role for OSCC carcinogenesis [12].

On the other hand, the oral cavity itself has one of the richest microbiotas among mucosal surfaces [14,15]. The microbiota has been recognized as the largest repository of genetic information in the human body [16,17] and has been demonstrated to be significantly involved in a large spectrum of diseases of different natures (neoplastic, degenerative, immune, etc.). The aim of the present work is to review the available evidence about the involvement of the oral microbiota and of *Fusobacterium nucleatum* in OSCC carcinogenesis, to start to explore its potential role in the clinical setting either as a risk factor or as a modifier of behavior, prognosis and the response to different treatment modalities.

## 2. The Oral Microbiota

The oral microbiota is one of the most important and complex microbial communities in the human body and includes several hundred different species. The microbial ecology varies according to the distinctive oral biological niches, each with a peculiar environment that promotes the colonization of different microbes. Among these niches are the gingival sulcus, the tongue, the cheek, the hard and soft palates, the mouth floor, the throat, the tooth surface and the saliva [18]. These microbial communities include bacteria, fungi, viruses and protozoa. In the adult human oral cavity, bacteria are the most abundant including Gram-positive and Gram-negative species, and facultative or obligate anaerobes, which may colonize these different niches according to their oxygen requirements and interact with the mucosal cells and other bacteria or fungi. The most prevalent bacterial phyla are *Firmicutes*, *Bacteroidetes*, *Proteobacteria*, *Actinobacteria* and *Fusobacteria*. At the genus level, the most prevalent are *Streptococcus*, *Haemophilus*, *Leptotrichia*, *Porphyromonas*, *Prevotella*, *Propionibacterium*, *Staphylococcus*, *Veillonella* and *Treponema* [18].

Microorganisms inhabiting the oral cavity form structurally and functionally organized communities, attached to surfaces as biofilms, with interspecies collaborations that provide mutual benefits and ecologic stability. Bacteria within a biofilm or attached to the mucosa communicate by producing, detecting and responding to small diffusible signal molecules, available in the oral environment or generated by other microorganisms, in a dynamic equilibrium which promotes oral microbiota homeostasis and a generally healthy condition [19]. The relationship between the oral microbiome and its host is dynamic, and many events during a person’s life can affect the balance of the species within these communities.

Though in the healthy mouth the composition of microbial communities is remarkably stable, several factors can alter the oral microbiota including diet, smoking, geographical location, alcohol consumption, socioeconomic status, age, gender, smoke, oral hygiene, the use of antibiotics and genetic factors [20]. Significant changes in the oral microbiota composition may lead to imbalances and rupture of the above-mentioned homeostasis, which may pave the way for oral diseases such as caries or periodontitis that are characterized by the overgrowth of bacteria with pathogenic potential. As an example, a switch in the gingival sulcus from Gram-positive, facultative, fermentative microorganisms to predominantly Gram-negative, anaerobic, chemoorganotrophic and proteolytic organisms has been linked with the destruction of periodontal tissue [21]. Periodontitis is defined as an advanced inflammatory gingival disease caused by bacterial dysbiosis, often associated with poor oral hygiene, characterized by an abundance of species such as *Aggregatibacter actinomycetemcomitans*, *Porphyromonas gingivalis*, *Treponema forsythia*, *Treponema denticola*, *Prevotella intermedia*, *Prevotella nigrescens*, *Parvimonas micra*, *Campylobacter rectus* and *Fusobacterium nucleatum*. The abundance or dominance of any of these potentially pathogenic species, or the rise of a specific combination of these periodontal pathogens, is seldomly detected in the oral cavities of healthy humans, and its emergence may indicate a dysbiosis that may lead to disease [18,22]. There are many other pathological conditions affecting the oral cavity that implicate the role of dysbiosis and alteration of the oral microbiota [23]. Among these are all the stages leading to the development of OSCC, from hyperkeratosis to different degrees of dysplasia to carcinoma in situ, which are all often clinically associated with oral leukoplakia [24,25,26]. This role may be at least partly be attributable to the endogenous nature of oral infections and the potential regulatory influence of oral bacteria on inflammation. The dysbiotic microbiome may influence the tumor microenvironment and may contribute to tumor progression by sustaining chronic inflammation [24,25]. For instance, oral leukoplakia (OLK) is the most common pre-cancerous lesion of the oral epithelium, and about 10–15% of OLK may transform into malignant growth [27]. Molecular characterization of oral premalignant lesions identified host transcriptome signatures and a microbiome composition peculiar to these lesions and showing an association with OSCC. It has been proposed that the detection of these signatures in oral lesions may help in the diagnosis of cancer at an early stage [28]. Hence, the composition of the oral microbiota, and the prevalence of some species in certain anatomical niches, may promote slowly progressive alteration of mucosa homeostasis that, while not leading to overt acute disease manifestations, may trigger the emergence of other pathological conditions including premalignant lesions and cancer, as increasingly confirmed by recent data [24,26,29,30].

Differently from oncogenic viruses, few microbes directly cause cancer, but many seem to contribute to its emergence and progression, often acting through the host’s immune system [31]. The microbiota may influence carcinogenesis through different mechanisms such as the release of substances with carcinogenic properties, such as toxins and surface proteins that directly target the human cell membrane or that can gain access to the host cell cytoplasm and nucleus, where they can affect and eventually impair cell growth regulation [32]. Classical examples are the well-known *Helicobater pylori* factors such as the cytotoxins VacA and CagA [33]. CagA, a toxin directly secreted in the host cell cytoplasm through a Type IV secretion system, induces malignancy by perturbing host cell signaling pathways and cell proliferation [34,35].

Bacteria can also influence host homeostasis by acting as an anti-apoptotic agent. Apoptosis is a distinct mode of cell death that is responsible for the deletion of cells in normal tissues, and the removal of these cells is known to prevent tumor development [36]. Thus, any agent capable of inhibiting apoptosis may promote the atypical build-up of cancerous cells. There has been substantial evidence of oral pathogens suppressing apoptosis and potentially promoting carcinogenesis; for instance, *P. gingivalis* activates Jak1/Akt/Stat3 signaling to control intrinsic mitochondrial apoptosis pathways [37,38].

However, accumulating evidence indicate that the stimulation of chronic inflammation seems the most common way through which bacteria can promote carcinogenesis, and bacteria capable of causing chronic infections are those most widely implicated [39]. Although inflammation is a natural immune response to protect the host, persistent bacterial infections may sustain local inflammatory responses that may promote the alteration of normal cellular homeostasis, genomic instability and mutations [4]. Inflammation begins with the recruitment of phagocytes to the site of infection, where they start secreting proinflammatory cytokines, such as TNFα and other chemokines and cytokines that attract more phagocytes and other cells of the immune system, further sustaining or amplifying the local inflammatory response. These cells respond through physiological processes mediated by cellular enzymes such as NADPH oxidase, superoxide dismutase (SOD), myeloperoxidase and nitric oxide synthase (NOS), which promote the release of reactive oxygen and nitrogen oxide species (ROS and RNOS). These free radicals and their secondary byproducts can damage DNA, proteins and cell membranes and indirectly induce cell repair. Thus, the stimulation of renewed cell division mounts leading to mutations, deletions or translocations as damaged DNA promotes the development of cancer cells [40]. Some bacteria release endotoxins, such as lipopolysaccharides, which can activate the inflammation-associated cytokine production that is the major factor in bacteria-induced inflammation and a contributor to carcinogenesis [41]. As an example, the carcinogenic potential of *H. pylori* is supported by molecules and proteins other than VacA and CagA, including peptidoglycan and LPS, which during chronic persistent infection continuously stimulate inflammation, thereby establishing a pro-carcinogenic environment.

A similar mechanism has been proposed for several bacterial species commonly inhabiting the oral cavity. For example, *Porphyromonas gingivalis* and *Fusobacterium nucleatum* (*Fn*) are commonly associated with the oral mucosa and can induce the production of inflammatory cytokines, cell proliferation, the inhibition of apoptosis, cellular invasion and migration, and finally genomic alterations and cancer [42].

### 2.1. Fusobacterium Nucleatum and Cancer

*Fn* is an obligate anaerobic Gram-negative bacillus belonging to the *Fusobacteria phylum*, named after its slender shape and spindle-like tips at both ends. It is mainly found as commensal in the human oral cavity and gastrointestinal tract, though translocation from the mucosa to the bloodstream may cause acute and severe infections, highlighting its pathogenic potential [43].

The genus Fusobacterium includes other species of interest for humans, such as *F. necrophorum* and *F. periodonticum* [44]. *Fn* is further divided into subspecies which show significant genetic variability, as recently highlighted in several genomic studies [45] that are prompting a revision of the current nomenclature [46]. The currently recognized subspecies are *Fn subsp. nucleatum*, *Fn subsp. polymorphum*, *Fn subsp. vincentii* and *Fn subsp. animalis*. Although these subspecies have been historically considered as functionally interchangeable in the oral cavity, direct evidence based on the fine identification of these subspecies is still unavailable [47]. The most studied *Fn subsp. nucleatum* is seldom detected in the oral cavity, while the most frequently isolated ones are *Fn. animalis* and *Fn. Polymorphum*, and studies are currently underway to investigate whether and which *Fn* subspecies predominate in health and disease. Furthermore, recent genomic analysis indicates that *Fn. animalis* may be further separated into two distinct clades (*Fna* C1 and *Fna* C2) showing different prevalence, anatomical niches and carcinogenic potential, at least in colorectal cancer (CRC) [48].

*Fn* gained enormous interest in relation to its oncogenic role in CRC and other tumor types [49]. More specifically, the role of *Fn* in CRC has been well investigated, and an accumulating body of experimental data is starting to delineate the mechanism of action and the prognostic consequences of *Fn*’s presence in the tumor. The first evidence emerged in Castellarin et al., who demonstrated an enrichment of *Fusobacterium* sequences in CRC compared to adjacent normal tissue [50]. A study carried out in China analyzed fecal microbial communities in healthy individuals and CRC patients and identified a remarkable increase in *Fn*-specific DNA sequences in the CRC cohort compared with the healthy control group [51]. Similarly, a North American study found that *Fn* was more abundant in stool samples from patients with CRC than in those from healthy subjects [52,53]. Indeed, it has been suggested that determining *Fn* loads may help predict clinical outcomes in CRC patients [54], indicating a potential prognostic role of *Fn*.

**Figure 2:**
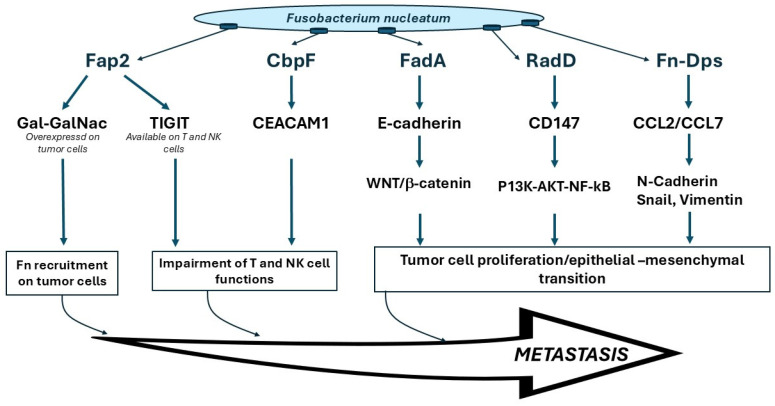
Main mechanisms involved in *Fusobacterium nucleatum*-induced carcinogenesis.

The physiological and molecular mechanisms responsible for the proposed carcinogenic activity of *Fn* have puzzled scientists for more than a decade but recent findings are shedding some light on the bacterial components, host receptors and targets involved (Figure 2). The mechanisms through which *Fn* is able to adhere to and invade the host cells is mediated by several adhesins (Aid1, CmpA, Fap2, FomA, FadA and RadD) localized on the *Fusobacterium* surface [55] that promote microbial co-aggregation and host-cell invasion and facilitate bacterial spread. The binding of *Fn* to epithelial cells occurs through specific recognition by these adhesins of host receptors. For example, Fap2 (fibroblast activation protein 2) attaches to the oligosaccharide Gal-GalNAc, which is overexpressed on cancer cells, enhancing the tropism of the bacterium for these cells and thereby explaining its enrichment in CRC [52]. Furthermore, Fap2 binds to and activates the T-cell immunoreceptor with Ig and ITIM domains (TIGIT), which is an immunoregulatory signaling receptor in T-cells and natural killer (NK) cells. This Fap2–TIGIT interaction protects both *Fn* and nearby tumor cells from the killing activity of NK cells, thereby serving as an inhibitor of immune responses in the tumor tissue that in turn further promotes the progress of CRC [56,57]. In addition, it is important to note that the anoxic and acidic tumor microenvironment is a suitable milieu for the growth of the obligate anaerobe *Fn*, further strengthening the tropism of *Fn* tumor tissue.

Another important virulence factor is FadA, which plays a crucial part in fusobacterial attachment to host cells and has been identified as the primary adhesin that facilitates the connection with various Gram-positive bacteria that are the initial colonizers of the oral mucosa and biofilms [55]. FadA binds to VE-cadherin on endothelial cells and to E-cadherins on epithelial cells [58], activating the oncogenic pathway Wnt/β-catenin, leading to increased expression of transcription factors, oncogenes, Wnt genes and inflammatory genes, as well as the growth stimulation of CRC cells. Indeed, the presence of *Fn* in CRC has been associated with higher cytokine levels and an inflammatory microenvironment supportive of tumor progression [59]. *Fn*-Dps has been identified as another important multifunctional *Fn* virulence factor that can lyse and disrupt erythrocytes, enhances *Fn* survival in macrophages and promotes CRC metastasis [60]. The trimeric autotransporters adhesins also play a role in mediating the attachment to host cells, as in the case of CbpF, which binds CEACAM1 available on the surface of cancer cells and immune cells in the tumor microenvironment [61,62]. The functional characterization of these *Fn* factors lends experimental support to the role of *Fn* in carcinogenesis, and most specifically in the molecular and immunological mechanisms that support tumor proliferation and metastasis.

Among the relevant questions to address, it would be very important to determine the source of the *Fn* that reaches and accumulates in the CRC or other cancers. It has been suggested that *Fn* can be transmitted via hematogenous transmission, from its typical habitat in the oral mouth to any part of the human body, including the gut mucosa [55]. Indeed, it has been shown that *Fn* can translocate from the oral cavity during periodontal disease, gingival injury and bleeding and, by the transient hematogenous route, reach the colon [63]. In line with this hypothesis, the injection of *Fn* into the tail veins of mice resulted in an effective colonization of colorectal tissue by the injected *Fn* strain [64]. However, we know that *Fn* is commonly found in the gut microbiota, and it has been shown that some *Fn* strains have the potential to disrupt the colonic–mucosal barrier, opening the possibility for a gut origin of the *Fn*-associated cancers in CRC. Future studies are needed to properly characterize the mechanisms of *Fn* colonization in CRC and the origin of the cancerogenic *Fn* strains.

### 2.2. F. nucleatum and OSCC

Since *Fn* is one of the common inhabitants of the oral cavity and has been implicated in CRC, several studies attempted to investigate its potential role in the genesis of SCC, the most common malignancy of the oral cavity (OSCC) [55]. Seminal studies observed that tissues from patients with SCC of the tongue and floor of the mouth showed an increased presence of *Fn* in tumor tissues than in healthy tissues, suggesting a specific association with the development of carcinoma [65]. The first clear epidemiological evidence for an association of *Fn* with OSCC was shown in Yemeni patients, where among the several taxa found to be differentially abundant between OSCC cases and healthy controls, *Fn* was the most significantly abundant genus in the OSCC samples [66]. Such a significant increase in *Fn*, together with *P. gingivalis*, in cancer lesions, has been confirmed in another study, again in 2017, underlying that it might be correlated with oral cancer [67].

In 2018, Yost et al. carried out a meta-transcriptome analysis to profile mRNA expression in the oral microbiome in OSCC [68]. They focused their study on non-smoking and HPV-negative OSCC samples to reduce the variability due to other high-risk factors for OSCC. From the transcriptional perspective, only *Fn* was found to be metabolically hyperactive in the oral community of OSCC patients, expressing putative virulence factors in agreement with some previous studies. Microbiome analysis of tissue samples confirmed that *Fn* was the most significantly increased of the 41 dominant genera evaluated in OSCC of the buccal mucosa compared to the control group [69]. These findings were confirmed by other studies reporting a greater abundance of *Fn* in OSCC lesions compared to contralateral normal tissue [70,71].

These and other results seem to corroborate evidence on *Fn*’s ability to promote cellular proliferation and tumor progression and invasion in human epithelium, not only in colorectal epithelium but potentially also in the oral cavity, both in vitro and in animal models (Figure 2) [17,52,72]. In fact, the role of *Fn* in OSCC cell proliferation was investigated using in vitro and in vivo experiments, and the results obtained suggest that *Fn* accelerates the OSCC cell cycle by activating the E-cadherin/Beta-catenin pathway, which is essential in the regulation of cell adhesions and oncogenic signaling [73].

Three recently published studies have also examined the prognostic effect of the presence of *Fn* in OSCC. Neuzillet et al. found that the presence of *Fn* was associated with improved overall survival, relapse-free survival and metastasis-free survival in their merged OSCC cohort [16]. In line with these findings, Chen et al. found that *Fn* enrichment in HNSCC tumor tissues correlated with better cancer-specific survival and a lower relapse rate [74]. In contrast, other authors report that the presence of *Fn* in OSCC correlated with the enhanced spread of cancers cells, tissue invasion and metastatic potential, which inevitably correlated with poorer survival in early-stage HPV-negative tongue cancers, clearly indicating a worsened prognosis in patients with *Fn* [75]. Given these contradictory findings and the fact that in CRC and other cancers the presence of *Fn* is commonly associated with a poor prognosis [76], it is necessary to better understand the role of *Fn* in OSCC and the mechanisms involved in the pathogenicity and carcinogenesis.

Considering the remarkable *Fn* genomic plasticity and the diverse features of different *Fn* strains, clades or subspecies, it may be interesting to investigate whether the different subspecies have the same abilities in terms of adhesion, invasion and pathogenicity. Currently, there are five *Fn* subspecies, which show a different tropism towards different sites of the oral cavity and other body districts. For instance, *Fn polymorphum* is dominant within dental plaques, whereas *Fn animalis* dominates the abscess environment [47]. Moreover, genomic analyses reveal that *Fn animalis*, considered a single subspecies, is instead composed of two distinct clades (*Fna* C1 and *Fna* C2), which have a different prevalence in the CRC tumor environment compared to the healthy mucosa. Of these, only *Fna* C2 dominates the CRC tumor niche [48], highlighting the differential abilities to adhere and multiply in these cells. It remains to be determined whether similar features or differences between *Fn* subspecies, or at the clade level, play a role in the emergence of OSCC.

Given the recent evidence implicating *Fn. animalis* as the predominant subspecies associated with CRC tumors [77] and its likely origination in the oral cavity, an interesting study developed a PCR primer set that quickly and unambiguously discriminates between the four *Fn* subspecies and *F. periodonticum*, to investigate the different prevalence of each *Fn* subspecies in the oral cavity. In this study, *Fn polymorphum* and *Fn animalis* comprised the most prevalent fusobacteria in the oral cavity, exhibiting a distinct inversely proportional abundance between the plaque and abscess specimens. The results of this semi-quantitative genotyping approach suggested that niche-specific ecological preferences likely exist among oral fusobacteria, especially *Fn polymorphum* and *Fn animalis* [47]. Consequently, it would be interesting to understand if the same difference in terms of prevalence of one subspecies compared to another can also be found in the tumor microenvironment. Interestingly, heterogeneity in adhesion phenotypes and potential pathogenetic properties were identified in strains of *Fn polymorphum* isolated from malignant and non-malignant oral lesions [78]. Table 1 summarizes the main studies implicating *Fn* in OSCC.

Nevertheless, the relative paucity of experimental evidence coupled with the challenges associated with the isolation in culture of *Fn* and the lack of standardized molecular tests to detect *Fn* (many studies use different primers and probes to detect and/or differentiate *Fn* subspecies [47,79]) make it challenging to obtain a clear picture on the role of *Fn* in OSCC.

## 3. Research Questions for Translational Research on *Fn* and OSCC

The experimental evidence accumulated in the last few years highlights the potential role of *Fn* in promoting OSCC. However, there are important aspects that require deep investigations, and here are some research questions that we considered important to address.

### 3.1. Where Does the Association Between Fn and OSCC Come from? Is Fn a Cause or a Consequence of Oral Carcinogenesis?

*Fn* may be one of the factors promoting carcinogenesis in the oral cavity, as has been postulated and partly demonstrated for CRC. While the role of *Fn* in promoting carcinogenesis and tumor growth in CRC has been investigated, the role of *Fn* in OSCC remains to be elucidated. Characterization of the molecular events occurring following in vitro infection of cells representative of the oral mucosa or of different OSCCs, and comparison with the results obtained in CRC cells, will shed light on these processes. Moreover, using *Fn* of different subspecies or clades, or even anatomical origin, may also help us to understand whether the mechanism involved in these two types of cancers is similar or not. These results will also be useful considering the potential relevance of *Fn* in other tumors, including breast and gastric cancers [80]. The use of relevant animal models of OSCC may be instructive to investigate whether the inoculation of specific strains of *Fn* may promote or not promote tumor growth.

However, *Fn*’s presence in OSCC may well not derive from a carcinogenic role and instead be a consequence of the changes in oral microenvironment induced by the presence of an often bulky and necrotic cancer lesion, maybe interacting with a tumor associated with immune cells.

In both scenarios, *Fn* may have a role in the clinical management of OSCC, as explained below.

### 3.2. Are the Fn Subspecies or Clades in OSCC the Same as Those Promoting CRC?

The environmental conditions in OSCC and in CRC are quite different, at least in terms of the microbiota and oxygen concentration, and they may affect *Fn* replication and interaction with host cells. It would be interesting to investigate whether or not the *Fn* subspecies and clades associated with CRC are the same as those associated with OSCC and eventually characterize the genetic features of the OSCC-associated *Fn*. Culture and molecular-based studies carried out on OSCC tumors aimed at detecting and characterizing *Fn* using WGS will be decisive to answer this important question. The results of these studies may be used to tailor improved diagnostics and potentially immunological therapies. Moreover, similarly to what was recently observed for CRC with *Fn animalis* clades [48], the genetic characterization of the *Fn* strains isolated in OSCC may help us understand whether some strains show enhanced carcinogenic potential, identify the associated genetic and phenotypic features [47] and investigate the correlation between *Fn* status and the degree of differentiation of OSCC.

### 3.3. What Is the Consequent Clinical Meaning of Fn in OSCC? Different Translational Perspectives Can Be Hypothesized

1. *Fn* is a classical initiating or promoting agent involved in the early progression from dysplasia to cancer/transformation. In this case, the microbiota would become a target for prevention, and modifications of the microbiota in high-risk subjects could reduce the OSCC risk. In this case, *Fn* should already be abundant in preneoplastic lesions.
2. The microbiota is modified by the presence of cancer, and *Fn* abundance is a consequence of a tumor’s impact on the oral environment and saliva (deriving from necrosis and cellular lysis, tumor metabolism, etc.). In this case, the *Fn* load would be proportional to the tumor bulk and increase in bulkier, more locally advanced OSCC.
3. The microbiota is a direct disease modifier. Independently from its role as a potential risk factor, it could drive carcinogenic progression toward specific genotypes and therefore clinical phenotypes. In this case, characterization of the genetic alterations in different OSCCs and their association with *Fn* may help to dissect the role of *Fn* in tumor growth and metastasis [81].
4. The microbiota influences the immune response, and consequently tumor–host interaction and balance, and this would explain the specific impact described on nodal involvement progression and recurrence, as an effective immune response against tumor could reduce the nodal spread.

### 3.4. What Is the Prognostic Value of Fn in OSCC?

The determination of the prognostic value of *Fn* in OSCC may be of great interest from a clinical/translational perspective. As we have summarized, the results of the available studies are somewhat conflicting, though some studies consistently report an association of *Fn* with a reduced regional (nodal) spread (both at diagnosis and as recurrence after primary treatment). Node metastasis is notably the main single prognostic factor in OSCC and is to some extent not fully predictable based upon other clinical parameters (such as tumor volume or depth of infiltration). In fact, in general neck treatment is always recommended in cN0 cases. Independently from the role of *Fn* and its mechanisms, the possibility of predicting neck involvement and the risk of neck recurrence would have a huge impact on OSCC management as it could allow us to sensibly differentiate the approach to the neck, in terms of the surgical approach (e.g., avoiding elective neck dissection in cT1-2cN0 low-risk cases, and indicating a more comprehensive neck dissection in cN0 high-risk cases) and the indications for adjuvant treatment (e.g., adding concomitant chemo to adjuvant radiotherapy in pT3 and/or pN1 cases).

## 4. Conclusions

In summary, we have outlined the emerging role of *Fn* in tumorigenesis and provided a critical review of the evidence collected so far on the specific role of *Fn* in OSCC. Future studies shall be useful to characterize the molecular, cellular and immunological interactions between the oral mucosa and the oral and tumoral microenvironment and *Fn*, underlying the association between the microorganism and OSCC. Consequently, the histopathological, clinical and prognostic implications of these findings shall be carefully analyzed and investigated to fully exploit their translational potential.

## Figures and Tables

**Table 1:** Summary of key studies implicating *F. nucleatum* in OSCC.

Reference	Role	Major Findings
[66]	OSCC carcinogenesis	*Fn* was the most significantly abundant genus in the OSCC samples
[70,71]	OSCC carcinogenesis	A greater abundance of *Fn* in OSCC lesions compared to contralateral normal tissue
[16]	OSCC prognosis	The presence of *Fn* was associated with improved overall survival, relapse-free survival and metastasis-free survival in their merged OSCC cohort
[74]	OSCC prognosis	*Fn* enrichment in HNSCC tumor tissues correlated with better cancer-specific survival and a lower relapse rate
[75]	OSCC prognosis	The presence of *Fn* in OSCC correlated with enhanced spread of cancers cells, tissue invasion and metastatic potential that inevitably correlated with poorer survival in early-stage HPV-negative tongue cancers, clearly indicating a worsened prognosis in patients with *Fn*
[78]	OSCC carcinogenesis	*Fn subsp. polymorphum* is most abundant in malignant oral mucosa, and strains with high copy number of TVSS adhesin-encoding genes show highest adhesion to oral keratinocytes

## Data Availability

No new data were created.
